# The Immune Response to the Myxozoan Parasite *Myxobolus cerebralis* in Salmonids: A Review on Whirling Disease

**DOI:** 10.3390/ijms242417392

**Published:** 2023-12-12

**Authors:** Naveed Akram, Mansour El-Matbouli, Mona Saleh

**Affiliations:** Division of Fish Health, Department of Farm Animals and Veterinary Public Health, University of Veterinary Medicine, 1210 Vienna, Austria; naveed.akram@vetmeduni.ac.at (N.A.);

**Keywords:** myxozoa, *M. cerebralis*, immune modulation, rt-PCR, flow cytometry, innate immunity

## Abstract

Salmonids are affected by the economically significant whirling disease (WD) caused by the myxozoan parasite *Myxobolus cerebralis*. In the past, it was endemic to Eurasia, but it has now spread to different regions of North America, Europe, New Zealand, and South Africa. Among salmonids, rainbow trout is considered the most highly susceptible host. Upon entering to the host’s body, the parasite invades the spine and cranium, resulting in whirling behaviour, a blackened tail, and destruction of cartilage. The disease is characterized by the infiltration of numerous inflammatory cells, primarily lymphocytes and macrophages, with the onset of fibrous tissue infiltration. Several efforts have been undertaken to investigate the role of various immune modulatory molecules and immune regulatory genes using advanced molecular methods including flow cytometry and transcriptional techniques. Investigation of the molecular and cellular responses, the role of STAT3 in Th17 cell differentiation, and the inhibitory actions of suppressors of cytokine signaling (SOCS) on interferons and interleukins, as well as the role of natural resistance-associated macrophage proteins (Nramp) in WD have significantly contributed to our understanding of the immune regulation mechanism in salmonids against *M. cerebralis*. This review thoroughly highlights previous research and discusses potential future directions for understanding the molecular immune response of salmonids and the possible development of prophylactic approaches against WD.

## 1. Introduction

Fish, being a potential source of nutrition, play an important part in meeting global food demands [1]. Climate change has created favourable conditions for the parasitic lifecycle, posing a serious threat to aquaculture, including salmonid fishes [2]. Among these threats, myxozoan parasites, particularly *Myxobolus cerebralis*, is a major challenge for salmonids. It is the causative agent of the serious disease named as whirling disease (WD) [3,4,5]. Studies have suggested that *M. cerebralis* stands out as the most notable and economically impactful parasite, leading to an estimated loss of $35–60 million in the US alone [6,7]. While previously believed to be enzootic only in Eurasia, WD has now been detected in various salmonid-rearing regions across Europe, the USA, Canada, South Africa, and New Zealand [8,9].

Whirling disease affects multiple salmonid fishes with varying severity and disease onset [10,11]. Clinical signs can be observed in Chinook salmon (*Oncorhynchus tshawytscha*), brook trout (*Salvelinus fontinalis*), sock eye salmon (*Oncorhynchus nerka*), rainbow trout (*Oncorhynchus mykiss*), and brown trout (*Salmo trutta*) [10,12,13]. Although different studies have revealed clinical progression in brown trout, rainbow trout is considered the most highly susceptible host [14,15,16]. The clinical signs caused by WD include a blackened tail, whirling behaviour, and death in infected fish [17,18,19]. *Tubifex tubifex*, as an obligate invertebrate host, releases triactinomyxon spores (TAMs) [20], which invade fish through mucous cell openings in skin, gill epithelium, and the oral route [21]. Following penetration, the sporoplasm travels within the skin and gill epithelium, subsequently invading epithelial cells [22]. As a result of internal budding within a cytoplasmic vacuole, the sporoplasm produces primary and secondary cells [21]. These primary cells, containing vegetative nuclei and generative cells, are named as plasmodia. They migrate through the peripheral nerves to spinal cord and brain and reside in the cartilage, leading to destruction of the ossification processes in the entire skeleton and necrosis due to granulomatous inflammation [14,22]. This cartilage damage activates the fish host’s immune system [16].

The fish immune system comprises two main components: the innate and adaptive immune systems [23]. Innate immunity is activated by pathogens and serves as the host’s first line of defence against infections [24]. *Myxosporean* spp. often provoke minimal or no host responses [21,25]. In fact, it is believed that they employ such a manipulative strategy to evade the fish immune system, avoiding local immune responses and potential inflammatory reactions at the point of entry [26,27]. Specifically, *M. cerebralis* uses an immune-privileged pathway to invade the head cartilage via the central nervous system and peripheral nerves, where there is a minimum of immune response [21]. TAMs exhibit a higher affinity for rainbow trout compared to brown trout. On the other hand, brown trout triggers more protective immune mechanisms that contribute to disease resistance [14]. The attachment of TAMs is influenced by mucosal factors, and a cellular protective response can be observed through the presence of eosinophils in the root ganglia of infected fish [15].

Limited information is available regarding resistance and susceptibility mechanisms among *M. cerebralis* and fish host species [28]. However, recent investigations have used fluorescence-activated cell sorting (FACS) [29] to reveal certain aspects of cellular and cytokine responses against *M. cerebralis*, thereby enhancing our understanding of the factors underlying susceptibility and resistance to WD [29,30,31,32]. Previously, both innate and acquired immune mechanisms have been reviewed in myxozoans [33,34]. Various efforts have also been made to investigate the role of STAT3 in Th 17 cell differentiation [35] and to study the potential inhibitory actions of suppressors of cytokine signalling (SOCS1 and SOCS3) on IFNγ and IL-1β [30] during WD. Moreover, the expression levels of natural resistance-associated macrophage proteins (Nramp α and β) [28] have been studied to stimulate the immune systems. An effort has been made to silence *M. cerebralis* serine protease (MyxSP-1) within the *T. tubifex* host using short-interfering RNA techniques to disrupt the life cycle of *M. cerebralis* [36]. Nevertheless, a complete understanding of fish immune mechanisms against *M. cerebralis* remains to be uncovered [4,37]. Although, the aforementioned research studies have investigated the expressions of different immune related genes, offering insights into the role of these genes in immunity against WD, further exploration is still required to fully comprehend the whole function of the immune system of host in defence mechanism against WD. Therefore, in this review, we highlight the comprehensive immune dynamics of salmonids and techniques (FACS, siRNA) used in investigation against *M. cerebralis,* aiming to unveil possible novel approaches in the future to enhance disease management and create prevention strategies.

## 2. Clinical and Histopathological Changes in Diseased Fish

**Figure 1:**
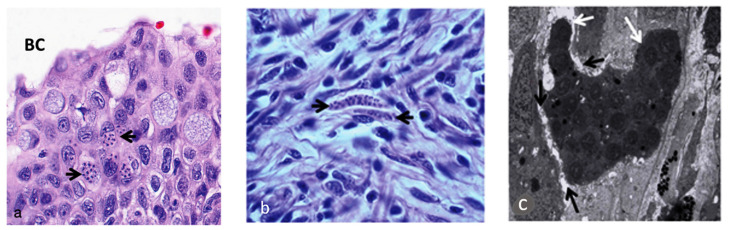
Presence of parasitic stages of *M. cerebralis* (black arrows) in buccal cavity mucosa, ×700 (**a**), and in nerve fibres and spinal cord, ×1050 (**b**) lesions of the epidermis, ×2800 (**c**) (Adapted from Sarker et al. 2015) [16].

**Figure 2:**
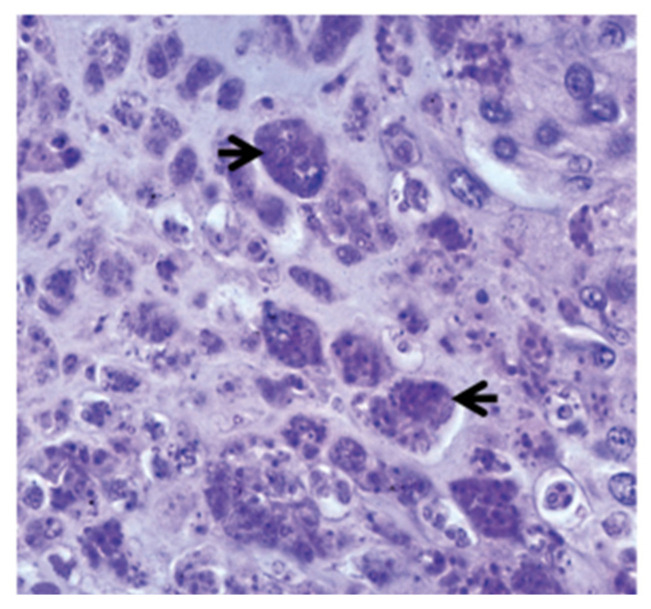
Aggregates of parasitic stages (black arrows) in the cartilage, 12 weeks post exposure, ×700 [16].

The parasite migrates to the spine and cranium, causing inflammation and lesions resulting in cranium destruction, which leads to damage and deformation of the skeleton in fish [38]. Moreover, it causes spinal cord constriction and brain stem compression, resulting in whirling behaviour and pressure on nerves, which control the pigment cells causing a black tail [8,39]. Numerous intra- and intercellular sporoplasm cells are found in the epithelia of the buccal cavity and epidermis (Figure 1a). *Myxobolus cerebralis* spores can also be found in gill arches [14]. Parasitic stages can be observed between the nerve fibres of the spinal cord (Figure 1b) [16], as well as in the head cartilage, vertebrae and gills [40]. The presence of cartilaginous necrotic foci rich in parasitic stages and vegetative cells characterizes the cranial lesions (Figure 2). The disease is characterized by the infiltration of numerous inflammatory cells, primarily lymphocytes and macrophages, with onset of fibrous tissue infiltration [41]. Reduced ability to maintain an upright orientation of the body was suggested to be due to the damage of the vestibular-auditory apparatus [42]. Following penetration through the integumentary system, the parasite attacks the nervous system directly, so scientists have focused more on immune studies in the cartilage and nervous system of salmonids.

## 3. Innate Immune Response

Two immune mechanisms are involved in fish immunity: innate immunity and adaptive immunity, also named the non-specific and specific immune systems, respectively [43]. Being efficient, innate immunity is considered the primary component in combating disease-causing pathogens in comparison with the acquired immune system [44]. Innate immunity is a rapidly responding strategy but does not deliver protection for long period of time [45]. The categorization of the innate immune mechanism is based on three compartments: the physical barrier and cellular and humoral factors [26,46].

### 3.1. Role of Physical Barrier in Immunity against M. cerebralis

**Figure 3:**
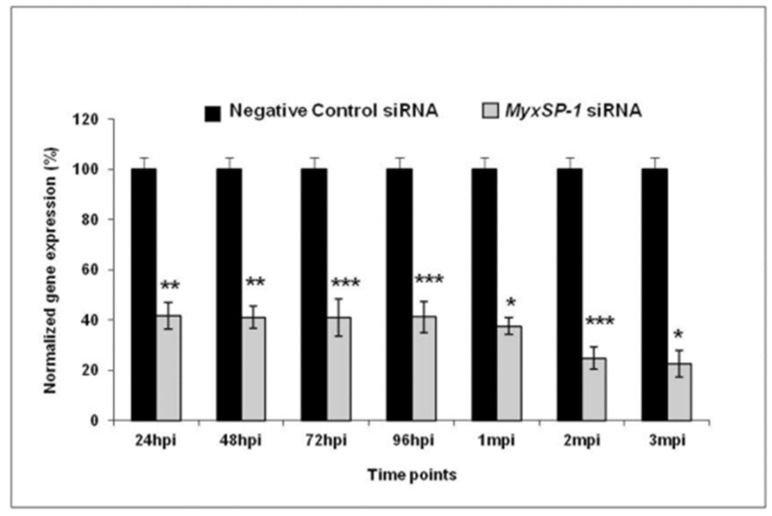
Normalized gene expression of MyxSP-1 gene after siRNA treatment of *T. tubifex* at different time points post infection (* *p* < 0.0001; ** *p* < 0.001; *** *p* < 0.005) [36].

The physical component of the immune system is a significant part of the innate immune system in fish. It includes scales, the mucous layer and epithelium present in the skin, gills and gastrointestinal tract, providing resistance to various infections [47,48,49]. Further, immune cells such as macrophages, lymphocytes, and eosinophilic granular cells are also found in the epidermis [47,50,51]. The triactinomyxon spores of *M. cerebralis* enter the fish body through these physical barriers [22,52] and encounter mucous barriers that consist of a complex of mucins containing lectins, lysozymes, C-reactive proteins, complements, haemolysins commensal microbiota, pentraxins and immunoglobulins (IgM) [53,54,55,56,57]. These biochemicals substances have biocidal or biostatic functions [58]. A study revealed the destruction of *M. cerebralis* within the cytoplasm of fish epithelial cells [21]. There is another report about presence of parasitic developmental stages in cytoplasm of phagocytes in the epidermis of the rainbow trout [59]. During the first few hours of intrapiscine development, *M. cerebralis* proteases were upregulated in the fins and gills after invasion. Genes encoding the serine protease (MyxSP-1) and cysteine protease (MyxCP-1) were assessed post infection. Upregulation in the expression of these genes was reported in the gills and dorsal fin tissues [60,61]. There was additional identification of the serine protease gene (MyxSubtSP) from *M. cerebralis* [62]. Sarker et al. [36] used short-interfering RNA (siRNA) to induce RNA interference (RNAi), targeting the MyxSP-1 for development of intervention strategy. The study silenced *M. cerebralis* MyxSP-1 in its annelid host *T. tubifex* via siRNA-induced RNAi. *T. tubifex* were infected with M. cerebralis myxospores and were subjected to treatment with MyxSP-1 siRNA or negative control siRNA, both at 2 μM concentration for 24 h at 15 °C. This treatment occurred at 24 h post infection (hpi), 48 hpi, 72 hpi, 96 hpi, 1 month post infection (mpi), 2 mpi and 3 mpi, respectively. After the final siRNA treatment at 3 mpi, the siRNA-treated *T. tubifex* were collected, and the expression of the MyxSP-1 gene was assessed using qPCR (Figure 3). When it was applied to rainbow trout fry, it prevented whirling disease and induced sustained RNAi in *T. tubifex*, representing a promising RNAi-based therapy for whirling disease in salmonids [36]. It would be beneficial to explore the optimization of siRNA methods and dosages to increase the efficiency of MyxSP-1 gene silencing in *T. tubifex*. Moreover, adapting RNAi therapy for diverse salmonid species and environments is essential for a comprehensive whirling disease strategy.

### 3.2. Cellular Immunity

Like mammals, the immune cell populations in fish include macrophages, lymphocytes, neutrophils, eosinophilic granular cells, basophils, and dendritic cells. Additionally, fish possess melanomacrophage centres and rodlet cells (RC) [63].

#### 3.2.1. Macrophages

Macrophages have a central role in immunity owing to their function in phagocytosis and lymphocyte activation. They have distinct receptors with the ability to recognize β-glucan, which allows immunostimulants to intensify leukocytes’ respiratory burst by producing reactive oxygen species possessing bactericidal properties [64]. In macrophages, nitric oxide is produced in ample amounts by inducible nitric oxide synthase iNOS, and assumes a pivotal role in the process of inflammation [65]. Previous studies have reported increased iNOS expression after *M. cerebralis* infection within progressive time periods of the disease. These observations were in the infected susceptible Trout lodge (TL) strain, while in the case of the resistant Hofer strain (HO), greater expression was evident only at 8 days post exposure (PE) [66]. Arginase is the distinctive enzyme involved in promoting the macrophage’s proinflammatory response [67]. There are two different isoforms of arginase, arginase-1 and arginase-2 [68]. In fish, arginase-2 is subjected to distinct regulatory mechanisms and is implicated in inducing an alternative activation state in fish macrophages [67]. A qRT-PCR-based study measured the expression level of arginase-2 and iNOS after exposure to *M. cerebralis* infection in rainbow trout. The susceptible fish strain TL exhibited increased levels of arginase-2 at 2 h, while in the case of resistant strain HO, the increase was observed 8 days after exposure [66]. The expression level of iNOS was upregulated in the susceptible strain from 24 h to 8 days post exposure, whereas in the resistant strain, this upregulation was observed only at 8 days post exposure. As a result of increased iNOS expression, it is expected that the inflammatory response and tissue damage will be more significant than strain H and strain T being unable to mount an effective immune response compared to resistant strains [69]. A research study compared the post-exposure expression level of Nramp α and β to *M. cerebralis*. Natural resistance-associated macrophage proteins (Nramp) have been considered a central figure in the innate immune response that arouses macrophage activity and boosts the macrophage’s capability to kill phagocytized pathogens. Downregulation of both genes Nramp α and Nramp β was noted in the vulnerable American TL strain at day 14 and day 40 following exposure, respectively [28]. This discovery indicated a possible Nramp involvement in the negative feedback mechanism [70]. Other studies have also reported similar gene expression in response to pathogens, but the cause of this regulation still needs to be disclosed [71,72,73]. Exploring the immune functional role of Nramp in the host through the regulation of the negative feedback mechanism would be a captivating endeavour.

#### 3.2.2. Lymphocytes

Lymphocytes are defensive cells analogous to B cells, T cells, macrophages, cytotoxic cells, and leukocytes [47,74,75]. Haematological responses against triactinomyxon spores of *M. cerebralis* have been investigated [76]. Lower numbers of lymphocytes were reported in infected fish. In salmonid fish, the presence of lower number of lymphocytes has been reported in *Saprolegina*-infected brown trout [77], in rainbow trout infected with *Vibrio anguillarum* [78], and post exposure to copper [79]. Physiological processes leading to stress [80,81], alterations in dynamics of lymphocytes due to the direct interaction of the fish immune system, lymphocyte destruction by pathogenic agents, and migration of lymphocytes from peripheral blood to invaded tissue are multiple factors involved in the cause of lymphopenia [78,82].

The modulation of immune cells in salmonids to combat *M. cerebralis* infection is summarized in Table 1. Densmore et al. (2004) reported lower activity of lymphocytes in *M. cerebralis*-infected rainbow trout against bioactive proteins, pokeweed mitogen, lipopolysaccharides, phytohemagglutinin, and concanavalin [83]. On the contrary, fish infected with *M. cerebralis* exhibited higher bactericidal activity of anterior kidney macrophages against *Yersinia ruckeri* than in noninfected fish. Because of the involvement of leukocyte suppression, functional increase, and the characteristics of the immune response evoked by the pathogen and secondary pathogen, difficulties will be encountered in extrapolating these results to other parasites and pathogens, especially on *M. cerebralis* [83]. However, a recent study revealed that proper activation of T and B lymphocytes in the head kidneys (HK), caudal fin (CF), and spleen (SP) allows more regulated immune cell responses and immunity against parasites in resistant HO rainbow trout strain [29].

#### 3.2.3. Granulocytes

Fish granulocytes are traditionally referred to with the terms neutrophil, basophil, and eosinophil. However, other terms such as eosinophilic granulocytes or fine granulocytes have also been used [86]. In mucosal infections (skin, gills, and intestines), granulocytes and phagocytes are typically the most prevalent immune system cells [63].

A wide range of hosts and variability in host susceptibility to *M. cerebralis* are evident [15,22]. It is thought that brown trout’s resilience compared to other salmonid hosts [84,87] is a result of this host’s evolutionary relationship with *M. cerebralis* in its native European habitat [88]. However, assuming that co-adaptation is the only cause of resistance based solely on geographic proximity is difficult to reconcile with the robust resilience of indigenous Trout lodge species like coho salmon [15]. Despite the fact that precise defence mechanisms remain incompletely elucidated, the existence of eosinophilic granular leucocytes in the root ganglia of afflicted brown trout, as opposed to rainbow trout, suggests the potential for a cellular protective response against the parasite [58].

#### 3.2.4. Mast Cells

Being a part of natural immunity, mast cells are found close to the skin’s blood vessels, gills, gastrointestinal tract, and ovaries [89]. These cells are identified using electron microscopy as exhibiting numerous cytoplasmic electron-dense granules. Various proteins like desmin, CD117, and S100 proteins are expressed by mast cell granules [90]. A number of factors, notably chronic inflammation and infection by parasites, lead to the induction of mast cells in fish tissues and organs [91].

A study concluded that coho salmon are immune to infection and the emergence of symptoms associated with WD [10]. Coho salmon, unlike rainbow trout or chinook salmon, which are susceptible to *M. cerebralis* infection, exhibited an abundance of eosinophilic granule cells (EGC) or mast cells in parasite-induced lesions or ganglia containing parasitic stages [85]. Brown trout species that are mildly resistant to *M. cerebralis* have shown similar mast cell responses [84,86]. For this reason, EGCs were suggested to have a role in the immunity of salmonids to whirling disease, but in general, their role is still unresolved [92], and detailed investigation could be helpful for future endeavours.

#### 3.2.5. Rodlet Cells

Rodlet cells are recognized by their distinctive thicker capsule-like cell borders and the presence of rodlet cytoplasmic inclusions resembling rods, and are closely related to inflammatory cells (eosinophile granule cells, mesothelial cells, epithelioid cells) [93] involved in the response against myxosporea [94,95]. Most often, these cells are found within the cardiac region, kidney, spleen, thymus, skin, gills, pancreas, gall bladder, and blood vessel endothelium [89]. They are responsible for various functions, having a secretory defensive role [96] and carrying out pH control, osmoregulation, electrolyte, and ion transport. They are mostly observed during parasitic infections, and can be triggered by any form of tissue damage, which ultimately stimulates leukocyte reaction due to chemotactic stimuli [95,97]. Disparity in the distribution of rodlet cells can be seen among various fish families. In salmonids, helminth infections are thought to be the reason for the induction of local recruitment of rodlet cells in affected epithelial cells [96]. Although some reports on rodlet cells and their defensive role in salmonids and other fish families against various parasites are available [98], the role of these cells during whirling disease still needs to be explored, and this would be fascinating development.

### 3.3. Humoral Immunity

Humoral immune parameters are a variety of soluble compounds that act as preventive agents by limiting the growth of microbes and neutralizing the enzymes on which the pathogen depends for pathogenesis [63]. Multiple nonspecific protective substances, including lectins, transferrin, antimicrobial peptides, and lysozymes, inhibit or suppress microbial growth [46]. In the case of myxosporean infections, multiple humoral innate factors such as lysozymes, peroxidases, and compliments are engaged in the eradication of pathogens [99]. During the development of whirling disease, parasites degenerate after entry into the host’s skin, and do not reach the peripheral nervous system [21]. Humoral immunity is thought to be involved in eliminating the *M. cerebralis* parasite from the fish’s skin, but the actual mechanisms involved should be interrogated [59].

### 3.4. Cytokine Response

Cytokines, being signalling low-molecular-weight secretory proteins, are considered regulators of the immune mechanism [31,100]. They are produced at the entry sites of pathogens to control phagocytes and neutralize entering microorganisms [101]. In general, fish have been found to possess various cytokines, including interleukin-1β (IL-1β), transforming growth factor-β (TGF-β), tumor necrosis factor-α (TNF-α), chemokines, and interferon (IFN) [102,103,104,105]. The IFN-γ related inflammatory response is modulated by SOCS proteins, and is involved in host’s response to *M. cerebralis* infection. Differential modulation of several interleukins (IL-17A, IL17-C, IL-21) and RORγ after *M. cerebralis* infection indicates the function of these molecules in rainbow trout immunity against the parasite (Table 2) [30]. Other studies have reported increased post-exposure expression of pro-inflammatory cytokines like IFNγ and IL-1β in brown trout and rainbow trout against *M. cerebralis* [35,106]. The increase in gene expression indicates their role in host protection against the infection [30]. The expression induction of TGF-β1b, SOCS1, and SOCS3 in brown trout following *M. cerebralis* exposure signifies their importance in mediating proper immune protection and restraining excessive inflammatory responses during the course of the infection [30].

The gene expression levels of SOCS1 and SOCS3 genes have been investigated following exposure to *M. cerebralis*. The parasite triggered the expression of SOCS1, IL-6-dependent SOCS3, IL-10, and Treg-associated transcription factor FOXP3 in the TL susceptible strain, which caused limited STAT1 and STAT3 stimulation, thereby impacting the Th17-mediated immune response [32]. The expression of SOCS1 and SOCS3 was instigated, which inhibits the stimulation of STAT1 and STAT3 in American TL strain, thus resulting in an imbalance of Th17/Treg17 and rendering the host incapable of launching a defensive reaction or regulating inflammatory responses, increasing vulnerability to WD. Conversely, within the resistant HO rainbow trout strain, the expression of SOCS1 and SOCS3 was controlled, while STAT1 and IL-23-mediated STAT3 expression enabled a more protective immune reaction. Fish immunity was promoted by the successful balancing of Th17/Treg17 responses, which was maintained by increased expression of STAT1 and IL-23-mediated STAT3. Therefore, the study demonstrated the key role of SOCS1 and SOCS3 in modulating the activation and significance of host immunity in rainbow trout [32]. Future investigations into the mechanisms of WD’s resistance to disease should focus on STAT3 and other factors influencing Th17/Treg cell distribution and balance [32,35].

## 4. Adaptive Immune Responses to *M. cerebralis*

The adaptive immune response in fish depends on T and B cells, as well as the diversity and specificity of their antigen receptors, which are called antibodies and T cell receptors, respectively [108,109]. For many years, it was believed that fish could not mount an adaptive immune response to myxosporea [110,111,112]. However, it has now been clearly confirmed that fish with various myxosporean infections, including whirling disease, have expressed particular antibodies [59,113,114].

### 4.1. T Cells

T cells are a type of lymphocytes that bear a surface T cell receptor, which identifies antigens in conjunction with MHC molecules [37]. Fish T cells include CD8+ cytotoxic T lymphocytes (CTL) and CD4+ T helper (Th) cells [37,115]. CTLs express the membrane bound glycoprotein CD8 and are capable of killing cells of the host that are infected [116]. Th cells express CD4+ molecules and release cytokines that control the activity of other cells of immune system. Fish CD4+ cells functionally differentiate into the effector subtypes Th1, Th2, Th17 and Treg [116,117,118]. By promoting CTL proliferation and macrophage activation, Th1 cells in fish facilitate the coordination of the immune response to ensure protection against intracellular infections [119]. Th2 cells aid in the promotion of B cell proliferation and antibody-mediated production, and are linked to immunity to external parasites [120,121]. Just like in mammals, in fish, Th1 and Th2 responses interact with IL4, lowering Th1 proliferation, and with IFNγ, hampering Th2 proliferation [122,123,124]. Th 17 cells are involved in mucosal immunity against extracellular pathogens including fungi and bacteria; they secrete IL17, IL21, and IL22 [117]. Regulatory T cells produce the anti-inflammatory cytokines IL10 and TGF, which help regulate the immune response [125].

Interferon-related genes (IFNγ and IRF1) were upregulated in rainbow trout infected with *M. cerebralis,* indicating an activation of the innate immune system in both strains. At 24 h and later time points, TL strain showed greater up-regulation of these two genes than the resistant HO strain [35]. This trend of increased transcription may be harmful to the susceptible TL strain, as it is necessary for IFNγ to keep a balance between anti-pathogenic effect and host inflammatory tissue damage [126]. In brown trout, the expression of IFNγ was greater in HK, SP, and CF. STAT3 expression was comparatively higher in the caudal fin of resistant HO fish, indicating it may induce resistance in the HO strain through activation of Th17 cells [35]. Th17 cells produce IL-17 and are thought to be the key player in resisting *M. cerebralis* at the epithelia [30]. Using flow cytometry, increased CD8+ and CD8− (presumably CD4+) T cells were observed in the CF, spleen, and HK in the resistant HO fish strain. The resistant strain exhibits a significantly more robust T cell response than the susceptible TL strain [29].

### 4.2. B Cells

Antibodies or immunoglobulins (IGs) are the primary elements of the immune response to infections [26]. Pathogen clearance through phagocytosis, virus and toxin neutralization, and complement cascade activation are a few of the immunological mechanisms mediated by IGs [127,128]. In teleost, the main three B cell lineages have been identified, resulting in the production of three different isotypes of immunoglobulins: IgM, IgD, and IgT/Z [129]. IgT was detected in rainbow trout and identified as IgZ in zebra fish [63]. Rainbow trout have expressed three subcategories of IgT. The IgT1 subclass has been reported in gut [130] and mucosal lymphoid tissues (gills) [131]. Similarly, IgT2 and IgT3 have been observed to be present in lymphoid organs and serum, respectively [130]. IgM has a role in systemic immunity, with IgM+ B cells predominating within both the blood and various systemic lymphoid organs, and during infection, their proliferation increases in the mucosal surface of skin and intestine [132,133].

Using flow cytometry, an increased cell count of IgM+ B was identified within the spleen, head kidney, and caudal fin in the *M. cerebralis*-resistant HO rainbow trout strain [29]. An experiment conducted by Ryce et al. (2003) investigated the acquired immune response of rainbow trout to *M. cerebralis*. It was determined that initial immunization exposure provides th fish with immunity to subsequent exposures [134,135]. Previously, a study detected antibodies against triactinomyxon spores’ antigen via Western blot and ELISA [135]. Samples from infected rainbow trout in the wild or in controlled laboratory experiments showed positive results using these assays. Fish that responded through antibody production to early stages of infection were subjected to Western blotting analysis, which demonstrated a varied antibody response without a regular or recurring pattern of antigen recognition. However, strong naturally acquired immunity is evident in reinfected rainbow trout, as these fish resisted the penetration of even a large number of spores. The acquired resistance was only found among formerly actively infected rainbow trout with cartilage lesions [59,135]. Regrettably, we have an insufficient understanding of the immunological mechanisms that resistant salmonids employ against *M. cerebralis*. Research studies on the innate immune response, as well as investigation of the humoral and cellular components of acquired immunity, can be planned to determine the elements involved in establishing acquired immunity.

## 5. Immune Modulation in WD-Resistant and Susceptible Fish

*M. cerebralis* can infect multiple species of salmonids [10,136,137]. Among the salmonids, brown trout is regarded as resistant, whereas rainbow trout is the most susceptible species and expresses serious disease consequences [10]. Coho salmon (*Onchorynchus kisutch*) is also considered a resistant strain, while the European Danube salmon (*Hucho hucho*) is highly vulnerable to WD [10]. It is not completely clear what causes the variable degrees of resistance shown in salmonids, and it seems that every species defends against the sickness through different mechanisms [15,84].

Severin et al. [66] evaluated the role of macrophages in the susceptibility of two different rainbow trout strains infected with *M. cerebralis*. The expression level of arginase-2 was noticeably more elevated in the susceptible strain TL than in the resistant strain Hofer (HO) at 2 h and 8 days post exposure. Moreover, the expression of iNOS was markedly induced at 24 h to 8 days post exposure in the susceptible American Trout lodge (TL) strain, and only at 8 days post exposure in the German strain HO. These findings suggested a low capability of the susceptible strain to regulate a successful immune response against infections with *M. cerebralis* [68]. Further, a study explored the dynamic transcriptional response of metallothionein and innate immune response genes to WD [107]. The obtained gene expression data elicited a more protective innate immune response of the Hofer strain than that of Trout lodge strain. The expressions of IFN-g, IL-1b, IRF1, and iNOS genes were higher in both susceptible and resistant rainbow trout after infection with *M. cerebralis*. In a different study, Nramp, as a candidate gene for resistance, was investigated in brown trout and rainbow trout after exposure to *M. cerebralis*. Reduced expression of Nramp α and β was evident in the Trout lodge strain compared to the resistant brown trout [28]. On the other hand, STAT3 was the only gene that showed significant upregulation in the German HO strain, while remaining consistent in the American TL strain [35].

In a preceding experiment, the gene expression profile was determined by microarray analysis and verified through qRT-PCR. Following exposure to *M. cerebralis*, the expression of ubiquitin-like protein 1 and interferon-regulating factor 1 was up-regulated 100-fold and 15-fold, respectively, in both rainbow trout strains. The expression of metallothionein B was increased over 5-fold in the resistant German HO strain compared to the susceptible American TL strain, wherein it remained unchanged. Metallothionein B is known to play a role in immune response and inflammation. On the other hand, the CC chemokine SCYA113 was increasingly expressed in the TL strain. The CC chemokine SCYA gene is a member of the CC chemokine family that directs leukocytes to areas of inflammation and infection [138]. The differential expression of these genes indicates that leukocyte migration to the infection site and their stimulation are crucial in determining fish’s vulnerability or resistance [107].

**Figure 4:**
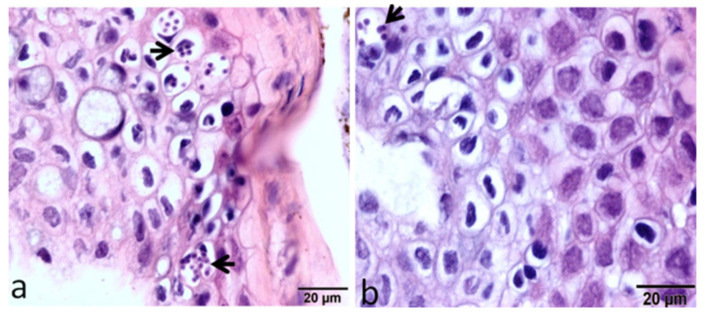
Large intracellular aggregates of the developmental stages of *M. cerebralis* appear in the epidermis of the susceptible host (**a**), while very few parasitic stages appear in the epidermis of the resistant strain (**b**) [29].

Flow cytometry-based research was conducted to investigate the dynamics of local and systemic immune cell responses in rainbow trout strains both susceptible and resistant to WD. A lower number of parasitic stages were noticed in the epidermis of the HO strain than in the TL strain at 12 h post exposure (Figure 4).

**Figure 5:**
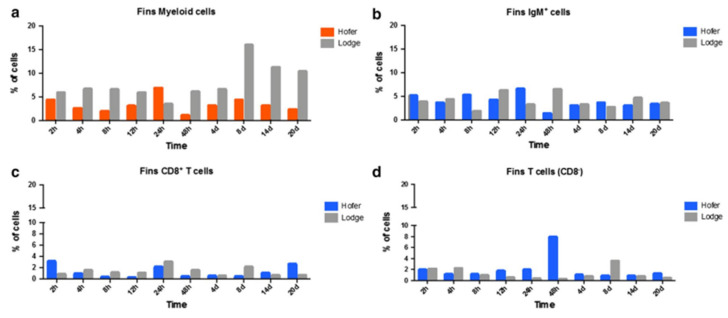
Flow cytometric analysis of HO and TL fish’s CF myeloid cells (**a**), IgM+ B cells (**b**), CD8^+^ T cells (**c**) and CD8^−^ T cells (**d**) [29].

In caudal fins (CF), myeloid cells showed increased levels only at 24 h post exposure in HO fish, whereas TL myeloid cells exhibited increased levels at all time points post exposure to *M. cerebralis*. The number of IgM^+^ B cells also increased in both resistant and susceptible fish at various time points during WD. Likewise, CD8^+^ and CD8^−^ T cells were also upregulated at multiple time points in both rainbow trout strains of *M. cerebralis*-exposed fish (Figure 5).

**Figure 6:**
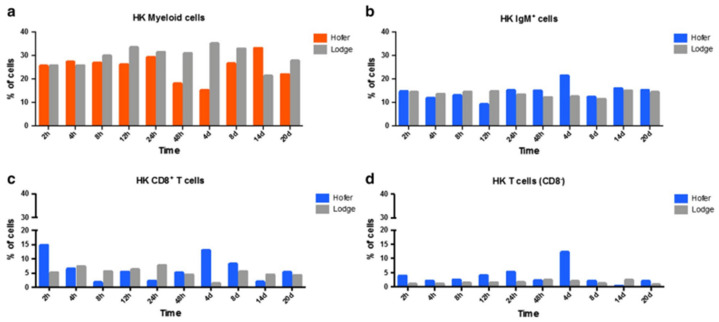
Flow cytometric analysis of HO and TL fish’s HK myeloid cells (**a**), IgM^+^ B cells (**b**), CD8^+^ T cells (**c**) and CD8^−^ T cells (**d**) [29].

**Figure 7:**
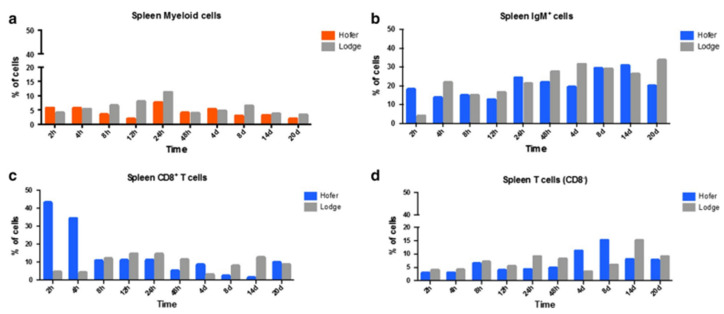
Flow cytometric analysis of HO and TL fish’s spleen myeloid cells (**a**), IgM^+^ B cells (**b**), CD8^+^ T cells (**c**) and CD8^−^ T cells (**d**) [29].

In the case of the head kidney (HK) and spleen of infected fish, the resistant HO strain elicited an increase in T cells and a decrease in myeloid cells compared to the susceptible TL strain. IgM^+^ B cells and CD8^+^ T cells were also markedly elevated in the HO strain compared to the TL strain. In the spleen, CD8^+^ and CD8^−^ cells were upregulated at various time points in the German HO strain, and at day 14 in the American TL strain (Figure 6 and Figure 7) [29]. The TL susceptible strain expressed excessive immune responses at all time points. The uncontrolled and excessive immune response in TL fish triggered irreversible inflammatory responses and tissue damage, favouring parasite development and contributing to host susceptibility [29]. Although our understanding of immune regulation has improved due to knowledge about immune response comparisons between susceptible and resistant strains, it would be useful to investigate the distribution and kinetics of regulatory and pro-inflammatory cells in both strains. Moreover, additional exploration of the cellular-based immune response implicated in WD would be helpful in disease exploration and ultimate disease prevention.

## 6. Immune Modulation in Response to Co-Infection

**Figure 8:**
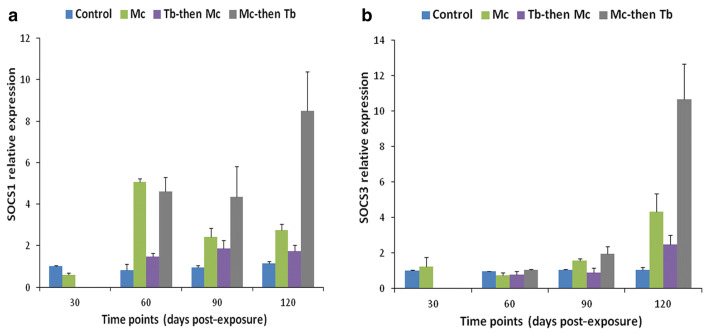
Relative gene expression of SOCS-1 (**a**) and SOCS-3 (**b**) in cranial cartilage during single infection and co-infections [141].

**Figure 9:**
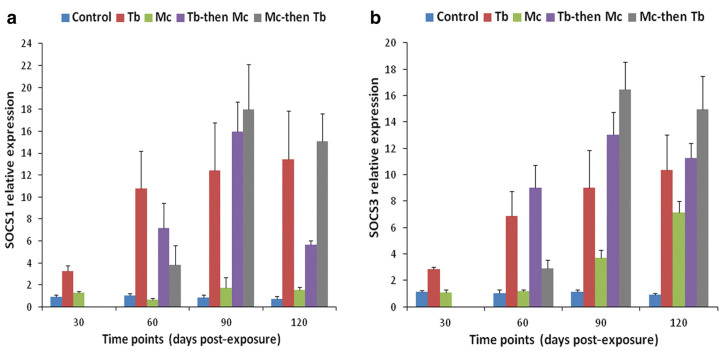
Relative gene expression of SOCS-1 (**a**) and SOCS-3 (**b**) in posterior kidneys during single and co-infections [141].

Co-infection has detrimental effects on the host, notably influencing their vulnerability to other infectious agents, the duration of the infection, and clinical progression [139,140]. An overview of comparative analysis of outcomes during whirling disease co-infection in salmonids is provided in Table 3. For instance, the immune modulation of rainbow trout exposed to *M. cerebralis* and *Tetracapsuloides bryosalmonae* was studied [141]. The host initially infected with *M. cerebralis* and then with *T. bryosalmonae* expressed greater numbers of parasites in both the posterior kidney and cranial cartilage, which are the target sites of *T. bryosalmonae* and *M. cerebralis*, respectively. The relative expression of the ribosomal protein L18 (RPL18) gene continued to rise, indicating parasitic activation. Moreover, the mortality rate was high, and upregulation of SOCS1 and SOCS3 was reported in both organs (Figure 8 and Figure 9).

**Figure 10:**
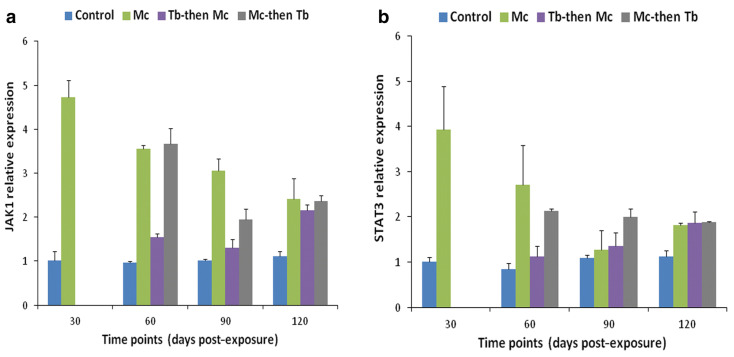
Relative gene expression of JAK-1 (**a**) and STAT-3 (**b**) in cranial cartilages during single and co-infections [141].

**Figure 11:**
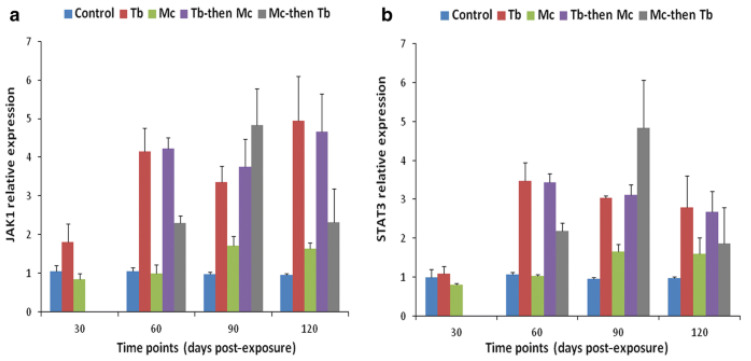
Relative gene expression of JAK-1 (**a**) and STAT-3 (**b**) in posterior kidneys during single and co-infections [141].

Likewise, elevated levels of JAK-1, and STAT-3 were also reported in both cranial cartilage and posterior kidneys (Figure 10 and Figure 11). The gene expression of SOCS1 and SOCS3 was much higher compared to JAK and STAT genes [141].

The synergistic effect established in the present case of co-infection was considered a result of *T. bryosalmonae*-mediated immunosuppression due to the downregulation of immune genes [141,142,143,144]. On the other hand, the fish group infected with *T. bryosalmonae* first and then co-infected with *M. cerebralis,* expressed a smaller number of both parasites. This was thought to occur due to the cross-reactivity between the sporogonic stages of both parasites, which led to cross immunity [145].

In another study, Densmore et al. (2004) reported the higher bactericidal activity of already *M. cerebralis*-infected rainbow trout against *Y. ruckeri* [83]. The greater bactericidal activity was due to the proliferative response of the immune system to *M. cerebralis* [146]. This represented an antagonistic interaction between the myxozoan parasite and bacterial pathogen. In the case of co-infection with primary infection of *M. cerebralis* followed by *T. bryosalmonae*, a synergistic effect was observed, resulting in more pronounced disease progression and mortality rate. However, in co-infection with *T. bryosalmonae* following *M. cerebralis*, less severe outcomes of the disease were noticed [41]. Hence, it was indicated that the consequences of co-infections depend upon the interaction between *M. cerebralis* and the co-infecting secondary pathogen.

## 7. Immune Modulation Due to Environmental Factors

As fish are poikilotherms, their physiology and body temperature are directly influenced by the ambient water temperature [147,148]. Immune response dynamics are linearly influenced by variations in water temperature due to changes in the season, salinity, microclimates, and fish migration [48]. Thermal stress can suppress the host immune system by altering the course of immune responses [48,149,150]. In a previous study, disease occurrence and the severity of lesions demonstrated a positive correlation with the increase in water temperature. This was due to the negative impact of temperature on fish immunity [14]. Another study investigated the contribution of bacterial pathogens, water temperature, and gas saturation in the mortality of rainbow trout fingerlings exposed to *M. cerebralis*. The increase in water temperature significantly increased mortality. Infection with *Flavobacterium psychrophilum* was only a significant issue when additional stressors were present, and the effect of gas supersaturation on mortality was negligible [151].

Furthermore, a study found a positive correlation between temperature and both the prevalence and mortality of *M. cerebralis* infection in rainbow trout [151]. Rainbow trout showed the highest prevalence of infection and the most serious lesions between 10 and 12 degrees Celsius. It is still not clear whether temperature-dependent illness variation in myxozoans, specifically *M. cerebralis* infection, is solely caused by adaptations in the immune system or by influences on the proliferation of myxozoans [152]. In addition, altitude can have an indirect impact on the immune mechanism in fish. A study was conducted to find a correlation between altitude and water temperature. A correlation between water temperature and altitude was identified. However, exceptions, such as low-altitude rivers fed by glacial water with consistently low temperatures, and high-altitude rivers with warmer temperatures due to surface water from shallow lakes, were observed. [153]. Based on the study mentioned earlier, it can be concluded that altering altitude influences water temperature, which can have effects on immune regulation. Makkula et al. (2007) observed that irradiated fish have decreased resistance to germs and parasites [154]. The impact of ultraviolet (UV) rays on the viability of the infective stage of *M. cerebrealis* in rainbow trout was evaluated. It was concluded that UV irradiation is effective in eliminating the infectivity of TAMs in fish [155]. Likewise, when juvenile rainbow trout were exposed to UV-treated TAMs, they did not inhibit epidermis attachment and penetration; however, they significantly hindered disease progression [156].

Stress is a further factor that has a major influence on the immune modulation of fish. Above all, corticosteroids and pro-inflammatory cytokines are potential factors causing such immunomodulation [157,158]. Increasing steroids in sea bream infected with *Ceratomyxa diplodae* [159] and *T. bryosalmonae*-infected rainbow trout caused increased susceptibility to their respective parasites [160]. In general, these studies suggested that stress has a negative impact on *M. cerebralis*-infected salmonids, but more research can help our understanding of the actual role of steroids in disease development.

Although studies have looked at the factors impacting morbidity and mortality, immune modulation in salmonids due to these effectors is still insufficiently understood. Hence, more research is needed to determine how stress, either external or internal, affects the immunological regulation of salmonids against *M. cerebralis* and other myxozoan parasites.

## 8. Future Perspectives

The distribution and recent spread of whirling disease [7,161], with its economic consequences on the aquaculture sector, particularly the trout farming industry, have significantly changed how scientists view *M. cerebralis*, the causative agent of whirling disease [3,59]. Although many innovative techniques such as siRNA, gene expression analysis, and FACS have aided in uncovering immune regulation, unfortunately, there are currently no available vaccines that can protect fish hosts from WD [16]. Given the lack of effective prophylactic strategies, the development of novel immunotherapeutics and effective vaccines is an increasingly urgent task. This review aims to enhance our understanding of immune modulation in salmonids in response to WD, and use of modern techniques. Emerging technical approaches like immunoproteomic analysis [162], computer-aided vaccine design using an in silico immunoinformatics approach [163], mRNA vaccines, and reverse vaccinology (subunit vaccines) [164] may help identify suitable targets for enhancing resistance and immunoprotection as a basis for disease prevention and management. Comparing immune responses, such as upregulation of CD8+ and CD8- (possibly CD+ T cells), and a significant increase in myeloid cells in the HO strain and the susceptible TL strain, respectively [29], can lead to a better understanding of immune modulation against the parasite. Investigating early skin interactions with the penetrating parasite might be helpful in determining the resistance traits of different salmonid species, and may provide valuable insights for future protective measures against WD. Furthermore, based on this review, functional studies and future research should address the knowledge gap regarding adaptive immune response. Specifically, the role played by various populations of B cells, T cells, and Treg cells should be investigated to understand how these cells shape resistance against WD. Such exploration may be valuable in developing effective future strategies to combat whirling disease in salmonids.

## Figures and Tables

**Table 1:** Summary of how immune cells are modulated in salmonids to combat *M. cerebralis* infection.

Immune Cells Studied	Fish Spp.	Immune Modulation	Techniques Used	Reference
Lymphocytes	Rainbow trout	Lower propagation of lymphocyte in *M. cerebralis* infected fish against bioactive proteins.	Bromodeoxyuridine (BrDU) incorporation assay	[83]
Granulocytes	Brown trout and rainbow trout	Eosinophilic granular leucocytes were noticed in the root ganglia of infected brown trout, but not of rainbow trout.	Histopathology and Immunohistochemistry	[84]
Mast cells	Chinook salmon, Coho salmon, and rainbow trout	Coho salmon, but not rainbow trout or chinook salmon susceptible to *M. cerebralis* infection, exhibited an abundance of many eosinophilic granule cells (EGC) or mast cells in lesions caused by parasites or ganglia containing parasitic stages.	Histological analyses	[85]
Eosinophilic granular leucocytes	Brown trout and rainbow trout	Eosinophilic granular leukocytes were more noticeable in brown trout compared to rainbow trout.	Histopathology and Immunohistochemistry	[84]
Myeloid cells, B cells and T cells	Rainbow trout	The study expressed that in the TL strain, there were overall increases in CF, HK, and SP myeloid cells, alongside drops in B cells and T cells in the SP and HK at carious time periods. Conversely, the HO strain primarily experienced an increase in T cells across CF, HK, and SP at various times.	FACS	[29]

**Table 2:** An overview of the expression patterns of immune-related genes involved in the immunity of salmonids to *M. cerebralis*.

Cytokines Studied	Fish Spp.	Immune Modulation	Technique Used	Reference
Ubiquitin-like protein, metallothionein B	Rainbow trout strains	After pathogen exposure, ubiquitin-like protein 1 exhibited a more than 100-fold upregulation, and interferon-regulating factor 1 demonstrated a more than 15-fold upregulation in both strains. The expression of metallothionein B was increased by more than 5-fold in the Hofer strain, whereas it remained unchanged in the Troutlodge strain after pathogen exposure.	qRT-PCR	[107]
Serine protease (MyxSP-1) and cysteine protease (MyxCP-1)	Rainbow trout	Post-infection upregulation of these genes in gills and dorsal fins exposed the enzymatic action in host tissue	qRT-PCR	[60,61,107]
Nramp α & β	Rainbow trout and Brown trout	A notable reduction in the expression of both genes was observed at various time intervals in the susceptible rainbow trout that were infected, in comparison to the noninfected cohort.	qRT-PCR	[28]
Arginase 2, iNOS	Rainbow trout	The expression level of both genes was upregulated in both the strains.	cDNA microarrays	[66]
iNOS	Rainbow trout	The susceptible American strain had increased expression only at one time point, while resistant HO strain had stimulation at two PE time intervals.	qRT-PCR	[35]
TGF-β	Rainbow trout	Resistant HO strain expressed more TGF- β in comparison to the susceptible TL strain.	qRT-PCR	[106]
IL-1β and IFN-γ	Brown trout	IL-1β and IFN-γ were upregulated in HK, SP, and CF at various time intervals post exposure to *M. cerebralis.* IL-1β increased during initial time points while IFN-γ elevated at initial and later times (including 2 dpe, when the lowest parasite quantity was detected).	qRT-PCR	[30]
IFNγ	Rainbow trout	Upregulation in *M. cerebralis*-infected rainbow trout and resistant fish showed a more rapid induction.	qRT-PCR	[35]
STAT3, IL-17A	Rainbow trout	In susceptible strains, the greatest expression of IL-17A was noticed in 2 dpe interacting with the highest parasite burden, in contrast to resistant strain.	qRT-PCR	[32]
KLF2, IL-1b, and innate immune response genes (IRF1, IFN-g, and iNOS)	Resistant (H) and susceptible (TL) rainbow trout	IFN-g, IL-1b, IRF1, and iNOS were upregulated post exposure to *M. cerebralis* for one or both strains over various time points. IFN-g and IRF1 showed a continuous increase in the TL strain in comparison to the HO strain. STAT3 was the sole gene with persistent elevated expression in the HO strain after infection, while remaining stable in the TL strain.	qRT-PCR	[35]
SOCS1 and SOCS3	Rainbow trout	The parasite induced the expression of SOCS1, IL-6-dependent SOCS3, IL-10 and Treg-associated transcription factor FOXP3 in a susceptible strain of rainbow trout, which caused limited STAT1 and STAT3 action, thereby having an effect on Th17 balance.	qRT-PCR	[32]

**Table 3:** Comparative analysis of whirling disease co-infection outcomes in salmonids.

Fish Spp.	Parasites	Co-Infection	Outcomes of Infection	Techniques Used	Reference
Rainbow trout	*M. cerebralis* and *T. bryosalmonae*	First, infection with *M. cerebralis*, followed by *T. bryoslamonae*	A greater number of parasites were observed in the posterior kidney and cranial cartilage. Upregulation of all immune genes (SOCS-1 and -3, JAK-1 and STAT-3) occurred in the kidney and crania. There was overexpression of SOCS1 and SOCS3 genes in the cranium. Increased expression of RPL18 was evident.	RT-qPCR	[141]
		Co-infection with *T. bryosalmonae* followed by *M. cerebralis*	There were a lesser number of parasites in infected organs. All immune genes showed higher expression, but this group elicited the downregulation of the RPL18 gene.		
Rainbow trout	*M. cerebralis* and *Y. ruckeri*	*M. cerebralis*-infected fish challenged with *Y. ruckeri*	The higher bactericidal activity of already *M. cerebralis*-infected rainbow trout against *Y. ruckeri* was observed.	Bromodeoxyuridine (BrDU) incorporation assay and Histological technique	[83]
Rainbow trout	*T. bryosalmonae* and *M. cerebralis*	Infection with *M. cerebralis* in conjunction with *T. bryosalmonae*	More severe pathological progression of each parasite with high mortality was observed. Along with more intense cartilage loss and displacement, a pronounced kidney swelling index of grade 4 was observed.	Histology and immunohistochemistry	[41]
		Infected with *T. bryosalmonae* concurrently with *M. cerebralis*	Typical pathological alterations associated with both parasitic diseases with a reduced mortality rate, similar to those caused by single *M. cerebralis* or *T. bryosalmonae* infection. Mild WD clinical signs without skeletal deformities were noticeable, and the kidney swelling index was grade 2 to 3.		

## Abbreviations

WD, Whirling Disease; HO, Hofer resistant strain of rainbow trout; TL, Trout lodge susceptible strain of rainbow trout; PKD, Proliferative Kidney Disease; SOCS, Suppressor of Cytokine Signaling; STAT, Signal Transducer and activator of transcription; TAMs, Triactinomyxon Spores; IFN, Interferon; Nramp, Natural Resistance-Associated Macrophage Proteins; IL, Interleukin; MyxSP, Myxobolus Serine Protein; MyxCP, Myxobolus Cysteine Protein; iNOS, inducible Nitric Oxide Synthase; HK, Head Kidney; CF, Caudal Fin; SP, Spleen; TGF, Transforming Growth Factor; TNF, Tumor Necrosis Factor; RC, Rodlet Cells; qRT PCR, quantitative real time polymerase chain reaction.
